# Tau PET With ^18^F-THK-5351 Taiwan Patients With Familial Alzheimer's Disease With the APP p.D678H Mutation

**DOI:** 10.3389/fneur.2019.00503

**Published:** 2019-05-22

**Authors:** Chin-Chang Huang, Ing-Tsung Hsiao, Chu-Yun Huang, Yi-Ching Weng, Kuo-Lun Huang, Chi-Hung Liu, Ting-Yu Chang, Hsiu-Chuan Wu, Tzu-Chen Yen, Kun-Ju Lin

**Affiliations:** ^1^Department of Neurology, Chang Gung Memorial Hospital, Taoyuan, Taiwan; ^2^College of Medicine, Chang Gung University, Taoyuan, Taiwan; ^3^Chang Gung Memorial Hospital, Molecular Imaging Center and Nuclear Medicine, Taoyuan, Taiwan; ^4^Molecular Imaging and Radiological Sciences, Chang Gung University, Taoyuan, Taiwan; ^5^College of Pharmacy, Taipei Medical University, Taipei, Taiwan

**Keywords:** amyloid, familial Alzheimer's disease, ^18^F-THK-5351, PET, Taiwan APP p.D678H mutation, brain ^18^F-AV-45

## Abstract

**Background:** Brain ^18^F-AV-45 amyloid positron emission tomography (PET) in Taiwanese patients with familial Alzheimer's disease with the amyloid precursor protein (APP) p.D678H mutation tends to involve occipital and cerebellar cortical areas. However, tau pathology in patients with this specific Taiwan mutation remains unknown. In this study, we aimed to study the Tau PET images in these patients.

**Methods:** Clinical features, brain magnetic resonance imaging/computed tomography (MRI/CT), and brain ^18^F-THK-5351 PET were recorded in five patients with the APP p.D678H mutation and correlated with brain ^18^F-AV-45 PET images. We also compared the tau deposition patterns among five patients with familial mild cognitive impairment (fMCI), six patients with sporadic amnestic mild cognitive impairment (sMCI), nine patients with mild to moderate dementia due to Alzheimer's disease (AD), and 12 healthy controls (HCs). All of the subjects also received brain ^18^F-AV-45 PET.

**Results:** The nine patients with sAD and six patients with sMCI had a positive brain AV-45 PET scans, while the 12 HCs had negative brain AV-45 PET scans. All five patients with fMCI received a tau PET scan with the age at onset ranging from 46 to 53 years, in whom increased standard uptake value ratio (SUVR) of ^18^F-THK-5351 was noted in all seven brain cortical areas compared with the HCs. In addition, the SUVRs of ^18^F-THK-5351 were increased in the frontal, medial parietal, lateral parietal, lateral temporal, and occipital areas (*P* < 0.001) in the patients with sAD compared with the HCs. The patients with fMCI had a significant higher SUVR of ^18^F-THK-5351 in the cerebellar cortex compared to the patients with sMCI. The correlations between regional SUVR and Mini-Mental State Examination score and between regional SUVR and clinical dementia rating (sum box) scores within volumes of interest of Braak stage were statistically significant.

**Conclusion:** Tau deposition was increased in the patients with fMCI compared to the HCs. Increased regional SUVR in the cerebellar cortical area was a characteristic finding in the patients with fMCI. As compared between amyloid and tau PET, the amyloid deposition is diffuse, but tau deposition is limited to the temporal lobe in the patients with fMCI.

## Introduction

The novel amyloid precursor protein APP p.D678H mutation (NM 000484.3 APP c.2032G > C) has been reported in two families with autosomal dominant Alzheimer's disease (AD) characterized by an early onset of memory impairment and progress to dementia, and a tendency to develop cerebral amyloid microangiopathy (1–3). The most characteristic findings in brain ^18^F-AV-45 (florbetapir) positron emission tomography (PET) include high amyloid deposition in the occipital and cerebellar cortical areas (3). In addition, the clinical progression is rapid, and dementia usually develops at 55–60 years of age. In a follow-up brain ^18^F-AV-45 PET study, an increased regional standard uptake value ratio (SUVR) was found in the brain cortical areas of patients with familial mild cognitive impairment (fMCI), but a slightly decreased regional SUVR was noted in patients with familial AD (4). The mechanisms of the biphasic course in the brain remain unknown. However, a saturation process may be responsible in patients with fMCI, and cortical atrophy and ventricular dilatation may be correlated with the decrease in SUVR in patients with familial AD as that in the world ADNI results (5, 6).

Alzheimer's disease is characterized by extracellular senile plaques composed of amyloid beta and intracellular neurofibrillary tangles consisting of paired helical filaments of hyperphosphorylated tau protein (7). The amyloid protein is considered to be a disease biomarker, while tau protein is considered to be a disease progression biomarker (8). The interaction between amyloid and tau protein remains unclear.

In this study, we aimed to investigate the tau deposition pattern in Taiwanese patients with fMCI with the novel APP p.D678H mutation as well as brain ^18^F-AV-45 PET to understand the effects of tau and amyloid protein in a specific family. We also compared ^18^F-THK-5351 PET findings among patients with fMCI, sporadic amnestic mild cognitive impairment (sMCI), sporadic AD (sAD), and healthy controls (HCs).

D678H mutation; VOI, volume of interest.

## Materials and Methods

### Participants

Ten symptomatic patients with familial AD were recruited from two Taiwanese families with the APP p.D678H mutation (3). Among them, five with MCI received brain ^18^F-AV-45 PET and ^18^F-THK-5351 PET scans. The other five patients with familial AD did not participate in this study, including four who had been admitted to a nursing home and could not tolerate the procedure and one who had died due to nasopharyngeal cancer. The medical history, neurological examinations, laboratory tests, and neuroimaging studies of the enrolled patients were reviewed. In addition, 27 patients including six with sMCI, nine with sporadic mild to moderate dementia due to AD (mild in 7, moderate in 2), and 12 HCs were selected for comparison. The 12 HCs were matched for age with the fMCI patients.

The patients with sAD were selected according to the following criteria: (a) a progressive course of memory impairment, (b) neuropsychological tests with the Alzheimer's Disease Assessment Scale-Cognitive subscale (ADAS-Cog), and (c) clinical dementia rating (CDR) score ≧ 0.5 (9). The patients with sMCI were selected based on the following criteria: (a) subjective memory complaints by the patient or an informant, (b) relatively normal performance on other cognitive domains, (c) normal activities of daily living, (d) objective memory impairment on at least one neurocognitive test of memory performance, and (e) no dementia according to DSM-IV criteria (10, 11). The exclusion criteria for this study were: (1) major systemic diseases such as severe heart disease, uremia, hepatic failure, myocardial infarction, poorly controlled diabetes, severe previous head injury, hypoxia, sepsis, and severe infectious diseases, (2) other major neurodegenerative disorders such as frontotemporal dementia, dementia with Lewy bodies, idiopathic Parkinson's disease, progressive supranuclear palsy, cortical basal syndrome, and spinocerebellar degeneration, (3) major cerebral vascular diseases, (4) implantation of metal devices such as a cardiac pacemaker or intravascular devices, (5) major psychiatric disorders including schizophrenia, major depression, drug or alcohol abuse, (6) pregnant women or breast feeding women, and (7) patients in whom MRI was contraindicated.

### Methods

Blood samples from all subjects were drawn for apolipoprotein phenotypes and biochemical studies including complete blood count, glutamic-oxaloacetic transaminase, glutamic pyruvic transaminase, blood urea nitrogen, creatinine, triiodothyronine, free tetraiodothyronine, thyroxine stimulating hormone, vitamin B12, folic acid, cortisol, and venereal disease research laboratory test (12). The HCs underwent the same procedures as the patients with fMCI, sMCI, and sAD. This study was approved by the Institutional Review Board of Chang Gung Memorial Hospital and the Ministry of Health and Welfare in Taiwan. The nature of research was explained to all of the subjects, and all signed informed consent forms. In addition, the next of kin or guardians of the patients with fMCI and sAD also gave written informed consent if the patients could not comprehend the study protocol or could not sign their names.

### Clinical Neuropsychological and Cognitive Assessments

All subjects (fMCI, sAD, and sMCI) underwent clinical and neuropsychological examinations. The detailed neuropsychological tests included the Mini-Mental State Examination (MMSE), CDR scale, Wechsler memory scale-revised, visual association memory test, category verbal fluency test, clock-drawing test, and trail-making A test, all of which were administered to obtain objective evidence of cognitive impairment (13).

### Brain MRI Procedure

All subjects received an MRI scan with a 3T MR scanner (magneton Trio, a TIM system, Siemens, Erlangen, Germany). The scanning protocol included an axial fluid attenuation inversion recovery (FLAIR) sequence (TR = 9,000 ms, TE = 87 ms, T1 = 2,500 ms, voxel size = 0.9 × 0.7 × 4 mm^3^) and whole brain axial three-dimensional (3D) T1-weighted magnetization prepared rapid acquisition gradient echo (MP-RAGE) sequence (TR = 2,000 ms, TE = 2.63 ms, T1-900 ms, flip angle = 9°, voxel size = 1 × 1 × 1 mm^3^), which was subsequently reformatted as planes perpendicular to the long axis of the hippocampus in slices 2 mm thick. An additional coronal T2-weighted turbo spin echo sequence (TR = 7,400 ms, TE = 95 ms, voxel size = 0.4 × 0.4 × 2 mm^3^) was acquired with identical geometric orientation with the reformed coronal T1-weighted images (12).

### Amyloid PET Acquisition

Radiosynthesis and acquisition of ^18^F-florbetapir PET images were performed as described previously (14). ^18^F-florbetapir PET scans were performed using a Biograph mCT PET/CT system (Siemens Medical Solutions, Malvern, PA). A 10-min PET scan was acquired 50 min after an injection of 375±18 MBq of ^18^F-florbetapir. The 3D ordered subsets expectation maximization reconstruction algorithm (four iterations, 24 subjects, Gaussian filter 2 mm, Zoom 3) was applied with CT-based attenuation correction and scatter and random corrections, which resulted in reconstructed images with a matrix size of 400 × 400 × 148 and a voxel size of 0.68 × 0.68 × 1.5 mm (15, 16).

### Tau PET Acquisition

Radiosynthesis and preparation of ^18^F-THK-5351 PET tracers were performed as described previously (17). Tau PET images were acquired 50–60 min after intravenous injections of 378 ± 17 MBq of ^18^F-THK-5351 on a dedicated PET/CT scanner (Siemens Biograph mCT 16; Siemens Medical Solutions). The same reconstruction protocols were applied as with the amyloid PET images. In addition, all subjects received an MRI scan with a 3T MR scanner (Magneton Trio, a TIM system, Siemens, Erlangen, Germany) to screen for other diseases and obtain structural information.

### Image Analysis

PMOD image analysis software (version 3.3; PMOD Technologies Ltd., Zurich, Switzerland) was used for all image processing and analysis. Each PET image was spatially normalized to the Montreal Neurological Institute space using MR-based spatial normalization. Eight volumes of interest (VOIs) were selected including the cerebellum gray, cerebellum white, frontal, medial temporal, lateral temporal, medial parietal, lateral parietal, and occipital areas, based on the anatomic labeling atlas (18). Because the fMCI patients may have had a high SUVR in the cerebellar cortical areas, the reference regions of cerebellum white and pons were used to calculate SUVR images for ^18^F-florbetapir and ^18^F-THK-5351, respectively. Regional SUVRs were measured from the mean SUVR of each VOI. For ^18^F-THK-5351 images, regional SUVRs for VOIs of Braak stage I/II, Braak stage III/IV, and Braak stage V/VI (19) were also computed for analysis.

### Statistical Analysis

Data are expressed as means **±** SD, or absolute numbers with proportions for descriptive statistics. Regional SUVRs of the ^18^F-Tau THK-5351 PET images were compared individually region by region using the non-parametric Kruskal-Wallis test with Dunn's multiple comparison *post hoc* analysis for group comparisons between the five patients with fMCI and 12 HCs, between the six patients with sAD and 12 HCs, and between the five patients with fMCI and nine patients with sAD. A *p*-value of 0.05 was considered to be the threshold for statistical significance in each test.

## Results

The demographic data and clinical characteristic of the five patients with fMCI from 2 large Taiwanese familial AD pedigrees are shown in Table 1 and Supplement Figure 1. There were two men and three women aged between 49 and 57 years. The age at onset of memory impairment ranged from 46 to 53 years, and the age at entry to the study ranged from 47 to 54 years. All five patients had memory impairment. The MMSE scores ranged from 23 to 26 (median: 24), and the sum score of CDR ranged from 0.5 to 5.0 (median: 3). The years of education ranged from 9 to 14 years.

**Table 1:** Demographic data of the patients with familial MCI and the Taiwan APP p.D678H mutation.

**Patient No**	**Gender/current age (Y)**	**Age at study (Y)**	**Onset age (Y)**	**Education (Y)**	**Clinical features**	**APoE**	**Brain MRI**	**Current diagnosis**
1	F/53	51	50	14	RMI	E3/E3	Multiple cerebral microbleeds^*^, CA	MCI
2	M/54	52	50	9	RMI	E3/E3	Normal	MCI
3	M/57	54	53	14	RMI	E3/E3	CA	MCI
4	F/49	47	46	12	RMI	E3/E3	Normal	MCI
5	F/54	52	52	14	RMI	E2/E3	Multiple cerebral microbleeds, CA	MCI, CAA

*Y, year; M, male; F, female; MCI, mild cognitive impairment; RMI, recent memory impairment; APoE, apolipoprotein; CA, cortical atrophy; MMSE, Mini-Mental State Examination; CDR, clinical dementia rating; MRI, magnetic resonance imaging; CAA, cerebral amyloid angiopathy*,

* *after 3 courses of passive immunization with aducanumab*.

**Figure 1:**
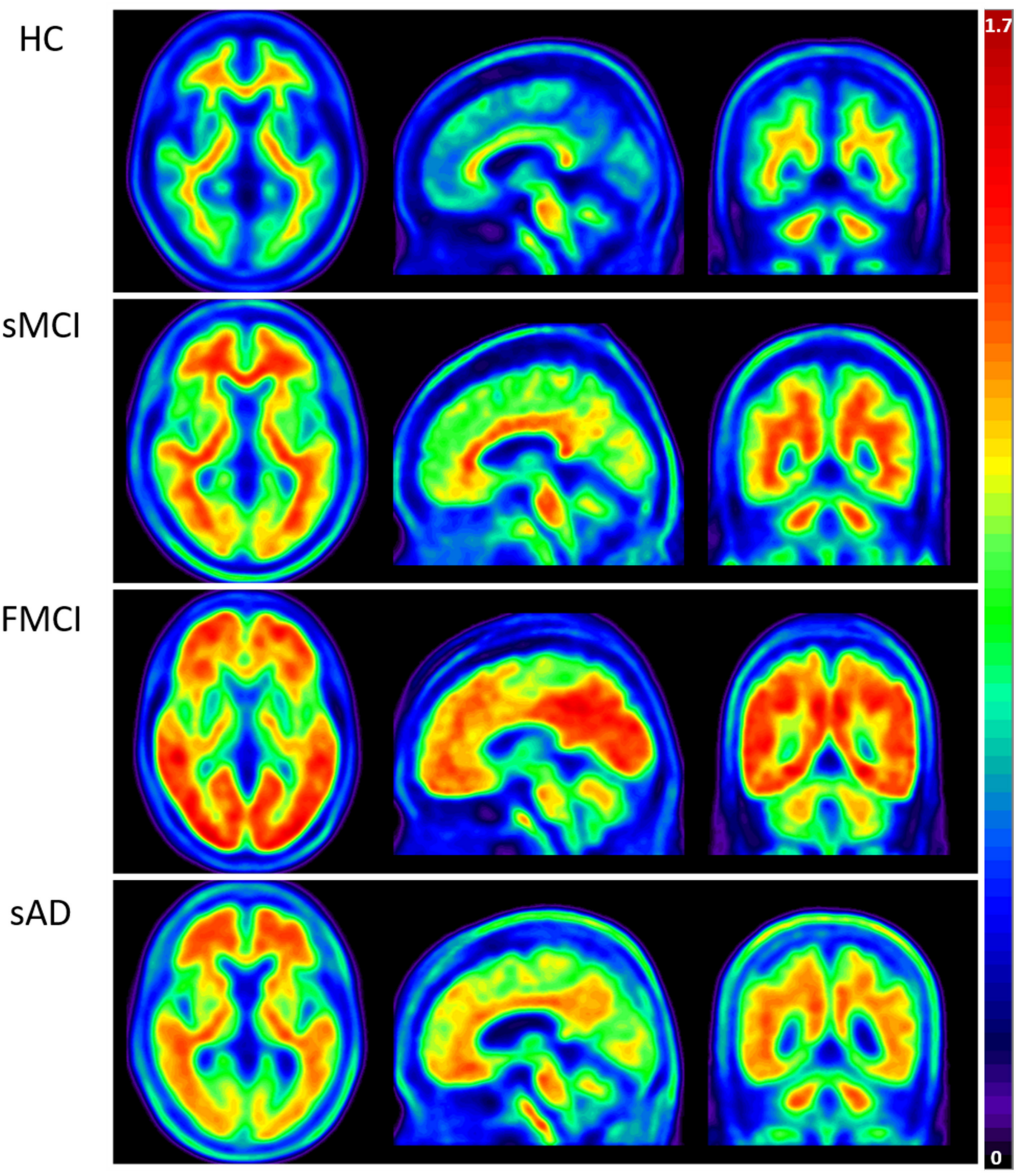
Group means of brain AV-45 PET images of the HCs and patients with sMCI, fMCI, and sAD. The figure shows prominent amyloid deposition in the frontal, temporal, parietal, precuneus, and particularly occipital cortical areas in the patients with fMCI, increased amyloid deposition predominantly in the precuneus and frontal areas in the patients with sAD and sMCI, and no definite uptake in the HCs.

**Figure 2:**
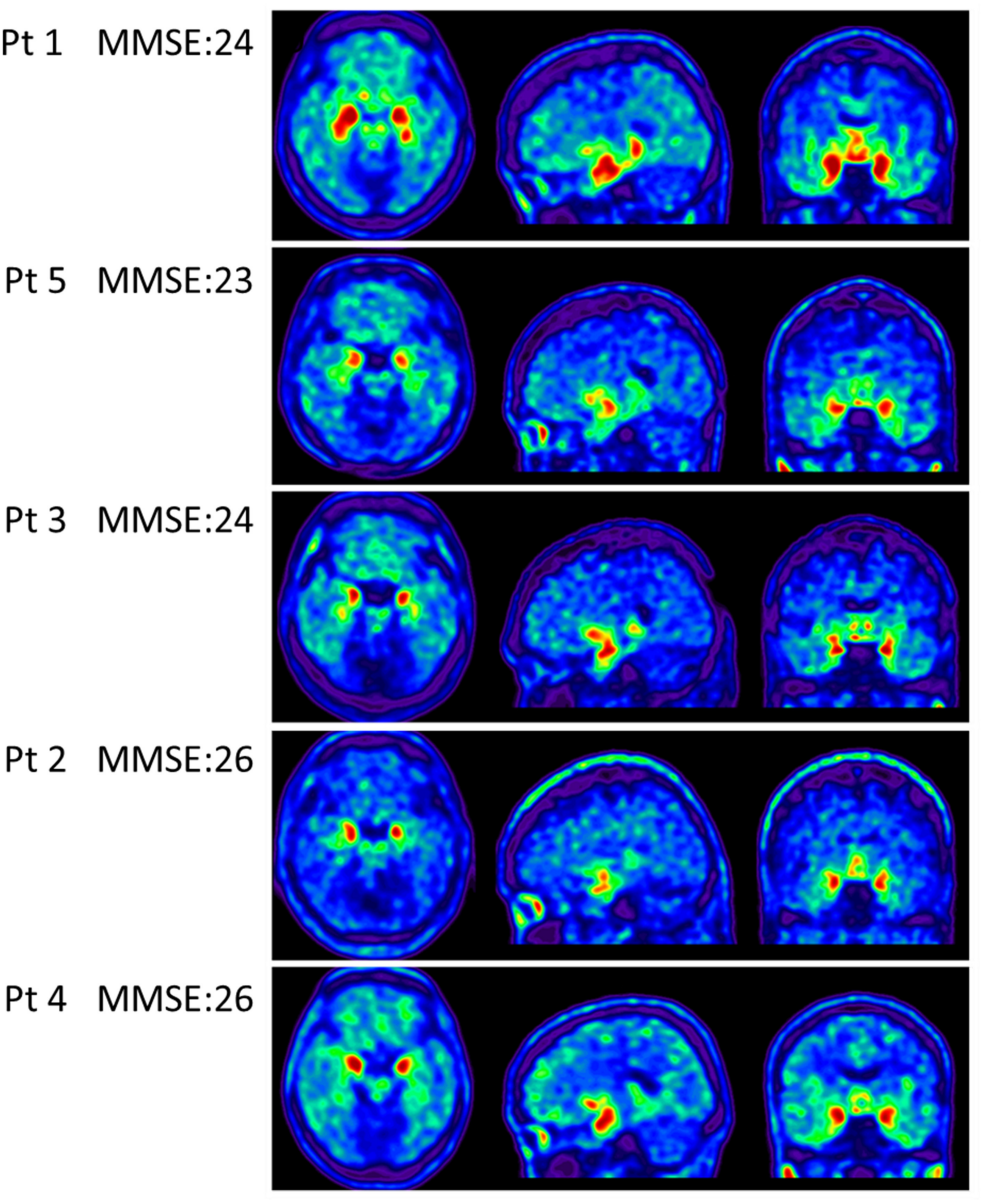
Tau PET scan with THK-5351 in the five patients with fMCI showing an increased SUVR of THK-5351 particularly extending to the lateral temporal area.

**Figure 3:**
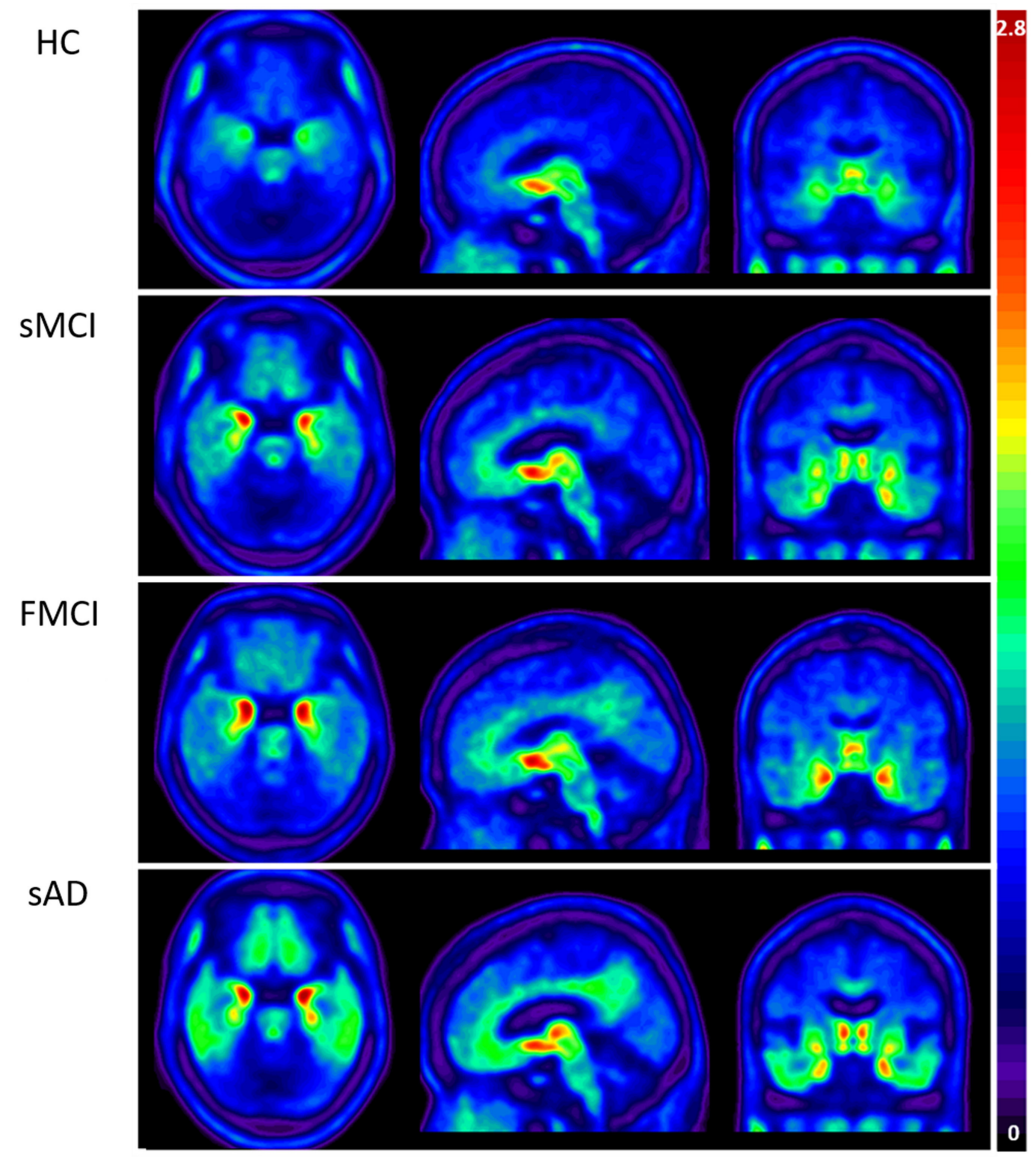
Group mean THK-5351 SUVR images of the HCs and patients with sMCI, fMCI, and sAD showing a trend of gradual uptake increase in the patients with sMCI, followed by those with fMCI and sAD.

Apolipoproteins E3/E3 were recorded in four patients with fMCI and E2/E3 in one. The brain MRI findings were normal in two patients (patients 2 and 4), and cortical atrophy was noted in three patients (patients 1, 3, and 5). Interestingly patient 1 had no microangiopathy initially, but after receiving three courses of passive immunization with a monoclonal antibody (aducanumab), she developed amyloid-related imaging abnormality-hemosiderin deposition (ARIA-H) with 0 to 3 microbleeds and subsequently up to 12 microbleeds. After stopping aducanumab treatment, the microbleeds remained stationary. The current diagnosis of the five familial patients was at the stage of MCI and cerebral amyloid angiopathy in one. Table 2 shows the demographic data of the five patients with fMCI, six with sMCI, nine with sAD (two: moderate, seven: mild), and 12 HCs. All of the fMCI patients had the APP p.D678H mutation, but none of the other groups had this mutation. All of the patients with sMCI, sAD, and fMCI had a positive AV-45 PET scan, while the 12 HCs had a negative AV-45 PET scan. In the patients with fMCI, brain AV-45 PET scans showed prominent amyloid deposition in the frontal, temporal, parietal, and precuneus, and particularly in the occipital cortical areas, while the patients with sAD and sMCI had increased amyloid deposition particularly in the precuneus and frontal areas (Figure 1). Figure 2 shows increased ^18^F-THK-5351 SUVR uptake in the five patients with fMCI, and in particular the THK-5351 uptake extended to the lateral temporal area. Figure 3 shows group means of THK-5351 SUVR data after 50–60 min. A trend of a gradual increase in THK-5351 SUVR was observed in the patients with sMCI, followed by those with fMCI and sAD. A significantly high regional THK-5351 SUVR was noted in the patients with fMCI and sAD compared to the HCs. In addition, high cerebellar cortex THK-5351 SUVR was observed in the patients with fMCI compared to the HC or sMCI groups.

**Table 2:** Demographic data of the patients with fMCI, sporadic AD, sporadic MCI, and healthy controls.

	**HC (*n* = 12)**	**sMCI (*n* = 6)**	**sAD (*n* = 9) mild:7, moderate:2**	**fMCI (*n* = 5)**
Gender	5M/7F	4M/2F	4M/5F	2M/3F
Age(Y)	50–72	70–89	57–79	47–54
Median	56.5	75.5	74	52
Mean ± SD	58.6 ± 6.8	78.5 ± 7.3	69.8 ± 8.5	51.2 ± 2.6
Education(Y)	6–18	6–16	6–16	9–14
Median	12	13	9	12
Mean ± SD	11.8 ± 3.4	12.3 ± 3.4	10.2 ± 3.9	12.2 ± 2.0
MMSE	23–30	21–28	3–21	23–26
Median	28	25.5	16	24
Mean ± SD	28.0 ± 1.9	24.7 ± 3.0	13.3 ± 6.6	24.6 ± 1.3
CDR(Sum)	0	1.0–4.5	2–14	0.5–5.0
Median	0	2.5	5.5	3.0
Mean ± SD	0	2.7 ± 1.4	6.7 ± 3.7	2.6 ± 1.7
APP gene	GG	GG	GG	CG

*HC, healthy controls; sMCI, sporadic amnestic mild cognitive impairment due to Alzheimer's disease; sAD, sporadic Alzheimer's disease; fMCI, familial mild cognitive impairment; Y, year; M, male; F, female; MMSE, Mini-Mental State Examination; CDR, clinical dementia rating; APP, amyloid precursor protein; CG, with the D678H APP gene; GG, without the D678H APP gene; PET, positron emission tomography*.

**Figure 4:**
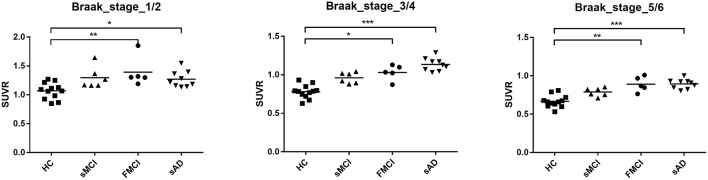
Regional SUVR of THK-5351 in the HCs and patients with sMCI, fMCI, and sAD according to Braak stage 1/2, 3/4, and 5/6. The regional SUVR of the patients with fMCI showed a significant increase compared to the HCs in Braak stage 1/2, and 5/6 (*p* < 0.01) and Braak stage 3/4 (*p* < 0.05). The regional SUVR in the patients with sAD had a significant increase in Braak stages 3/4 and 5/6 compared with the HCs (*p* < 0.001). *p < 0.05, **p < 0.01, ***p < 0.001.

**Figure 5:**
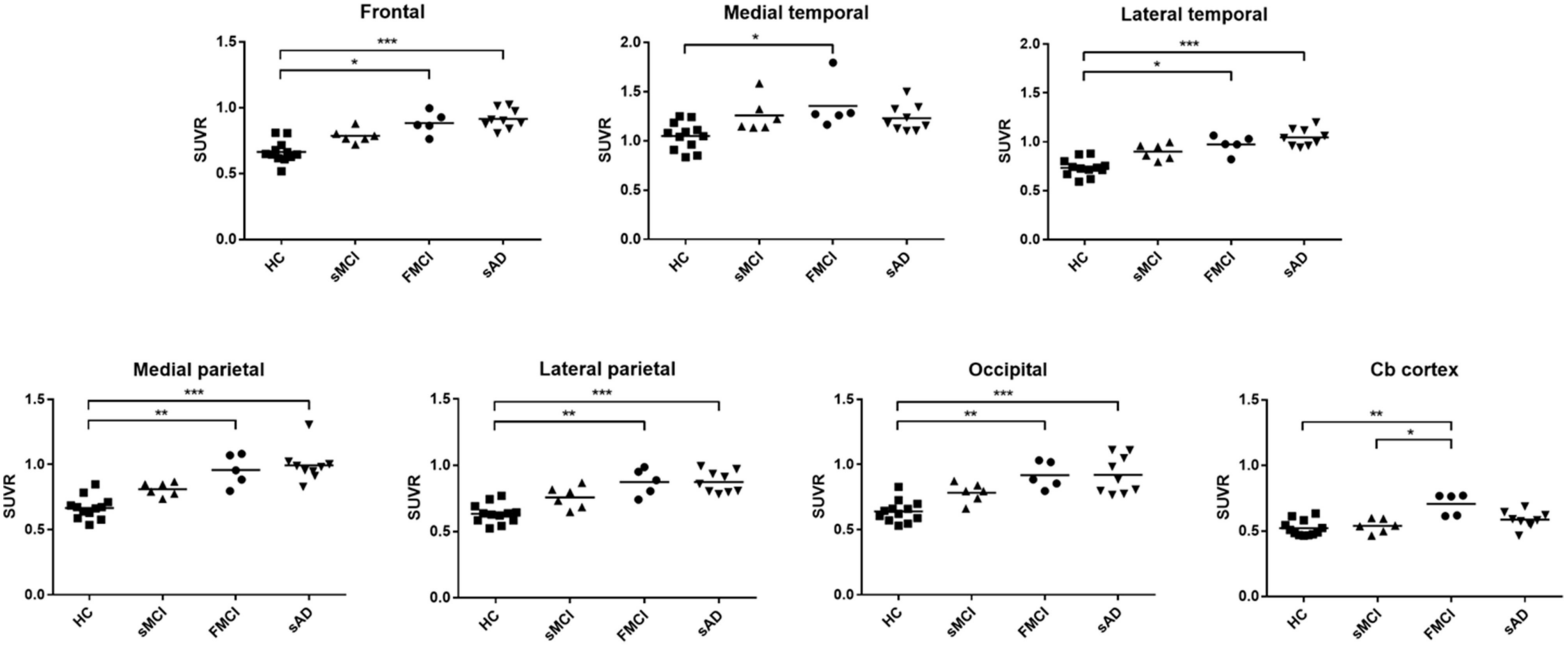
The regional SUVR of THK-5351 imaging in the HCs and patients with sMCI, fMCI and sAD. A significantly increased uptake was noted in the patients with fMCI in the frontal, lateral temporal, medial parietal, lateral parietal, medial temporal, occipital, and cerebellar cortical areas compared with the HCs, while a significantly increased uptake was noted in the frontal, medial parietal, lateral parietal, lateral temporal, and occipital areas in the patients with sAD compared to the HCs. In the cerebellar cortical area, the SUVR was increased in the patients with fMCI compared to those with sMCI. *p < 0.05, **p < 0.01, ***p < 0.001.

**Figure 6:**
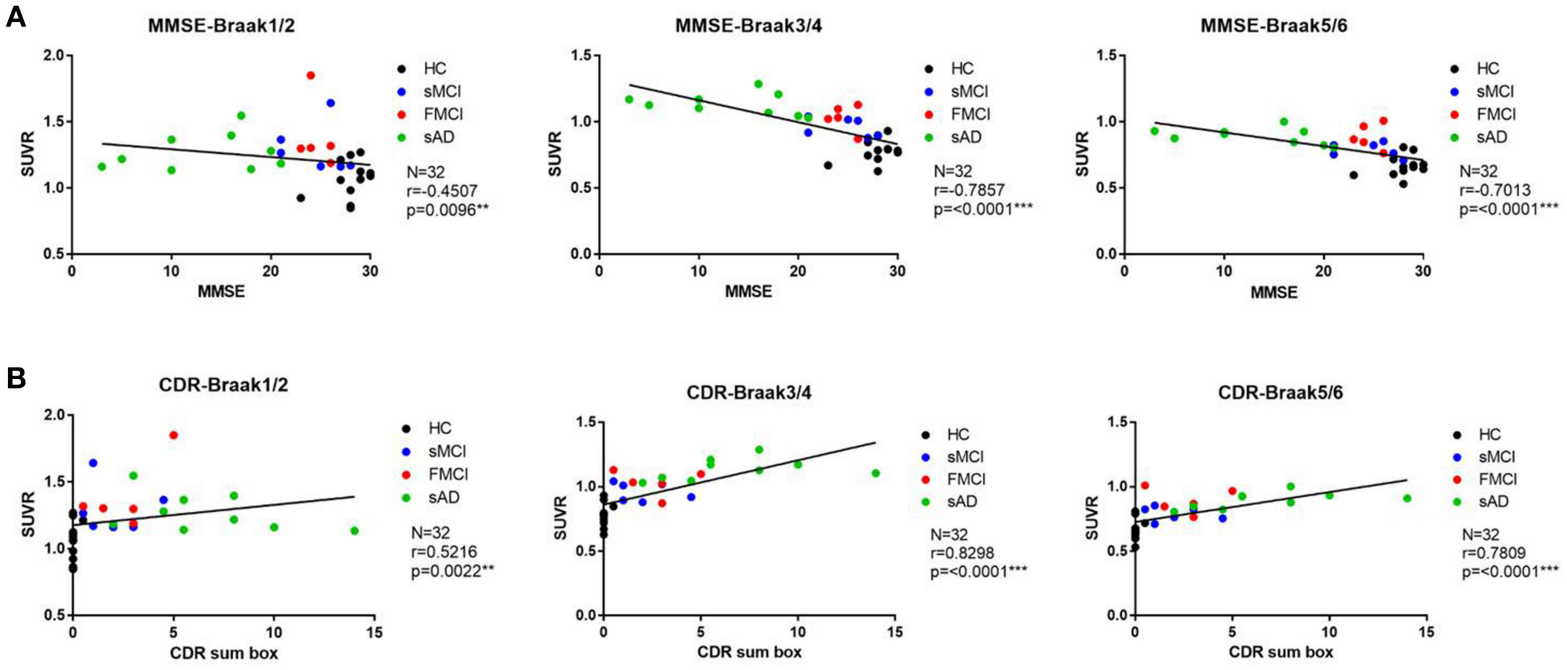
**(A)** The correlation between regional SUVR and MMSE within VOIs of Braak stages. Statistically significant correlations were noted in all VOIs of Braak 3/4 and 5/6 (*p* < 0.0001) and Braak 1/2 (*p* = 0.0096). **(B)** The correlations between CDR and regional SUVR within VOIs of Braak stages. A statistically significant correlation was noted in all VOIs of Braak 3/4 and Braak 5/6 (*p* < 0.0001) and Braak 1/2 (*p* = 0.0022).

Substantial ^18^F-THK-5351 uptake was noted in the medial temporal area in the HCs. According to Braak stage, the patients with fMCI had a significantly increased SUVR of ^18^F-THK-5351 than the HCs with Braak stage 1/2, and 5/6 (*p* < 0.01) and with Braak stage 3/4 (*p* < 0.05) (Figure 4). In the patients with sMCI, the regional SUVR of ^18^F-THK-5351 was not significantly increased compared with the HCs. However, in the patients with sAD, the SUVR was significantly increased in those with Braak stage 3/4 and 5/6 compared with the HCs (*p* < 0.001). There was no significant increase in ^18^F-THK-5351 SUVR between the patients with fMCI and sMCI and between those with fMCI and sAD (*p* > 0.05). With regards to regional SUVR of ^18^F-THK-5351, a significantly increased uptake was noted in the frontal, and lateral temporal areas (*p* < 0.05) and the medial parietal, lateral parietal, medial temporal, occipital, and cerebellar cortical areas (*p* < 0.01) in the patients with fMCI compared to the HCs. In addition, the regional SUVR of ^18^F-THK-5351 was increased in the frontal, medial parietal, lateral parietal, lateral temporal, and occipital areas (*p* < 0.01) in the patients with sAD compared to the HCs. In cerebellar cortical areas, the SUVR was increased in the patients with fMCI compared to the HCs (*p* < 0.01) and patients with sMCI (*p* < 0.05) (Figure 5). There was a statistically significant correlation between regional SUVR and MMSE score in all VOIs of Braak 3/4, 5/6 (*p* < 0.0001) and Braak 1/2 (*p* = 0.0096) (Figure 6A). There were statistically significant correlations between CDR sum box scores and regional SUVR in all VOIs of Braak 3/4, Braak 5/6 (*p* < 0.0001), and Braak 1/2 (*p* = 0.0022) (Figure 6B).

## Discussion

In this study, the patients with fMCI had a significantly increased SUVR of ^18^F-THK-5351 in the seven cerebral and cerebellar cortical areas (*p* < 0.01) compared with the HCs. In addition, the SUVR of ^18^F-THK-5351 in the cerebellar cortex was statistically significant compared to the patients with sMCI (*p* < 0.05). Despite a high level of tau deposition in the cerebellar cortex areas in our patients with fMCI, cerebellar symptoms, and signs were not found. Moreover, in the patients with sAD, the regional SUVR of ^18^F-THK-5351 in most of the cerebral cortical areas including frontal, medial parietal, lateral parietal, lateral temporal, and occipital areas was significantly higher (*p* < 0.001) compared with the HCs.

Furthermore, brain ^18^F-AV-45 PET showed an increase of amyloid deposition in the frontal, temporal, parietal, and particularly occipital areas in the five patients with fMCI compared with the HCs and patients with sMCI and sAD. These results revealed a higher amyloid burden than those in the patients with sMCI and sAD.

In the clinical course of disease progression, the patients with fMCI had an earlier onset of memory impairment and more rapid progression to dementia than the patients with sMCI (4). In addition, the patients may have had dementia and/or amyloid angiopathy (3). One patient (patient 5) who had multiple microbleeds in brain MRI compatible with cerebral amyloid angiopathy, cannot participate in the anti-amyloid treatment and another (patient 1) had no cerebral microbleed in the initial brain MRI but developed 12 microbleeds (ARIA-H) 3 months after passive immunization with aducanumab. Then she was withdrawn from the study. Although ARIA-H may also be found in other sAD patients who have received aducanumb therapy, these results may suggest a potentially higher risk of microhemorrhages during/after passive immunization in these fMCI patients because they may develop cerebral amyloid angiopathy.

However, tau deposition in these specific Taiwanese patients with the APP p.D678H mutation remains unknown. In this study, we clearly demonstrated tau deposition with THK5351 PET scan in the seven cerebral areas, and particularly the cerebellar cortical area compared with the HCs. However, the mechanisms of co-localization of amyloid and tau deposition remain unknown. Tau images may have shown a synergistic effect of burden on the brain in the patients with fMCI compared to the patients with sMCI.

According to the correlation between regional SUVR of ^18^F-THK-5351 and MMSE, there were statistically significant correlations within VOIs in Braak 1/2 (*p* = 0.0096), Braak 3/4 (*p* < 0.0001), and Braak 5/6 (*p* < 0.0001). In addition the correlations between regional SUVR and CDR sum box scores were also statistically significant in all VOIs of Braak 1/2 (*p* < 0.0022), Braak 3/4 (*p* < 0.0001), and Braak 5/6 (*p* < 0.0001). These results may indicate that ^18^F-THK-5351 uptake is correlated with disease severity.

In the patients with sMCI, there was a trend of increased SUVR of ^18^F-THK-5351 PET in all of the cerebral cortex areas, however the differences did not reach statistical significance compared with the HCs, which may be due to a small sample size. In the patients with sAD, the SUVR of ^18^F-THK-5351 PET was significantly increase in the frontal, medial parietal, lateral parietal, lateral temporal, and occipital areas (*p* < 0.001). This may indicate that ^18^F-THK-5351 PET can provide important clues for disease progression. In the patients with fMCI, there was statistically significant ^18^F-THK-5351 SUVR in the cerebellar cortex compared to the patients with sMCI. This may indicate that tau deposition in the cerebellar cortical area is a characteristic finding in patients with the Taiwan APP p.D678H mutation.

There are several limitations to this study, including that ^18^F-THK5351 binding may target a non-specific process related to neurodegeneration that is co-localized with tau pathology (20, 21). ^18^F-THK-5351 may also indicate a non-specific neuronal injury rather than specific tauopathy. The other important findings include off-target regions in the white matter, midbrain, thalamus, and basal ganglia (22). Increased ^18^F-THK-5351 uptake in the basal ganglia and substantia nigra may also reflect its binding to MAO-B (23, 24). Several different tau tracers such as flortaucipir (25), PBB3 (26), and PMPBB3 (27) have been developed and utilized in clinical studies. The recent study with PMPBB3 have been studied in patients with AD, progressive supranuclear palsy, frontotemporal dementia, and corticobasal degeneration (28). Further investigation with a more specific tau PET tracer such as PMPBB3 for the specific family is indicated. The other limitation is a small sample size of the fMCI patients, and a sMCI and sAD patients with relatively earlier onset and similar education level.

The interaction between amyloid and tau protein remains unclear. However, amyloid β plaques may enhance tau-seeded pathologies by facilitating neuritic tau aggregation (29). In addition, the amyloid β protein may promote the spread of tau through specific components of a neural system, leading to early symptoms of amnesia (30, 31). In this specific family with amyloid APP p.D678H mutation, a widespread of amyloid deposition was noted while a limited tau deposition in the medial and lateral temporal lobe areas is plaucible in patients with fMCI. The group difference of tau deposition between fMCI and sMCI is limited, although a trend is noted. The only difference is noted in the cerebellar cortex area. The real etiologies remained unknown. However, from the amyloid PET study, increased metabolic vulnerability in early onset AD is not related to amyloid burden (32). In our patients with fMCI, although increased SUVR of AV-45 PET in the occipital area, visual symptoms, or signs was not found. In this study the increased tau deposition in the cerebellar cortex area did not make any cerebellar dysfunction in patients with fMCI.

In conclusion, the present data indicate a higher tau burden in patients with fMCI with this specific Taiwan mutation. In addition a higher ^18^F-THK-5351 SUVR in the cerebellar cortical area was a characteristic finding in the patients with fMCI. ^18^F-THK-5351 PET may also be a biomarker of disease severity from sMCI to sAD. As compared between amyloid and tau PET, the amyloid deposition is diffuse, and tau deposition is limited to the temporal lobe in the patients with fMCI. Further investigations of the specific family members including asymptomatic heterozygotes and additional tracers are warranted. In addition, if the findings can be supported through cerebrospinal fluid examination and post-mortem neuropathology, we can understand the evolution of the familial dementia.

## Ethics Statement

The study was approved by the ministry of Health and Welfare in Taiwan and the Institutional Review Board of Chang Gung Memorial Hospital.

## Author Contributions

Y-CW, K-LH, C-HL, T-YC, H-CW, and C-CH: providing information. C-CH, I-TH, and K-JL: conception and design of the study. Y-CW, C-YH, and C-CH: acquisition of data. I-TH, K-JL, C-YH, and C-CH: analysis and/or interpretation of data. I-TH, C-YH, and C-CH: drafting of the manuscript. I-TH, K-JL, and C-CH: reviewing the manuscript critically. T-CY, K-JL, and C-CH: application for the funding. All authors approval of the final version of the paper.

### Conflict of Interest Statement

The authors declare that the research was conducted in the absence of any commercial or financial relationships that could be construed as a potential conflict of interest.

## Abbreviations

AD: Alzheimer's disease
APP: amyloid precursor protein
AV-45 amyloid: Amyvid
CCA: cerebral amyloid ongiopathy
CDR: clinical dementia rating
fMCI: familial mild cognitive impairment
HC: healthy control
MCI: mild cognitive impairment
MMSE: Mini-Mental state examination
MRI/CT: magnetic resonance image/computed tomography
sMCI: sporadic amnestic mild cognitive impairment
sAD: sporadic Alzheimer's disease
SUVR: standard uptake value ratio
PET: positron emission tomography
Tau tracer: 18F-THK-5351
Taiwan mutation: APP p.
