# Supplementary figures and images for: Tau PET With 18F-THK-5351 Taiwan Patients With Familial Alzheimer's Disease With the APP p.D678H Mutation
Source: Front Neurol. 2019 May 22;10:503. doi: 10.3389/fneur.2019.00503 (PMC6538951; doi:10.3389/fneur.2019.00503)

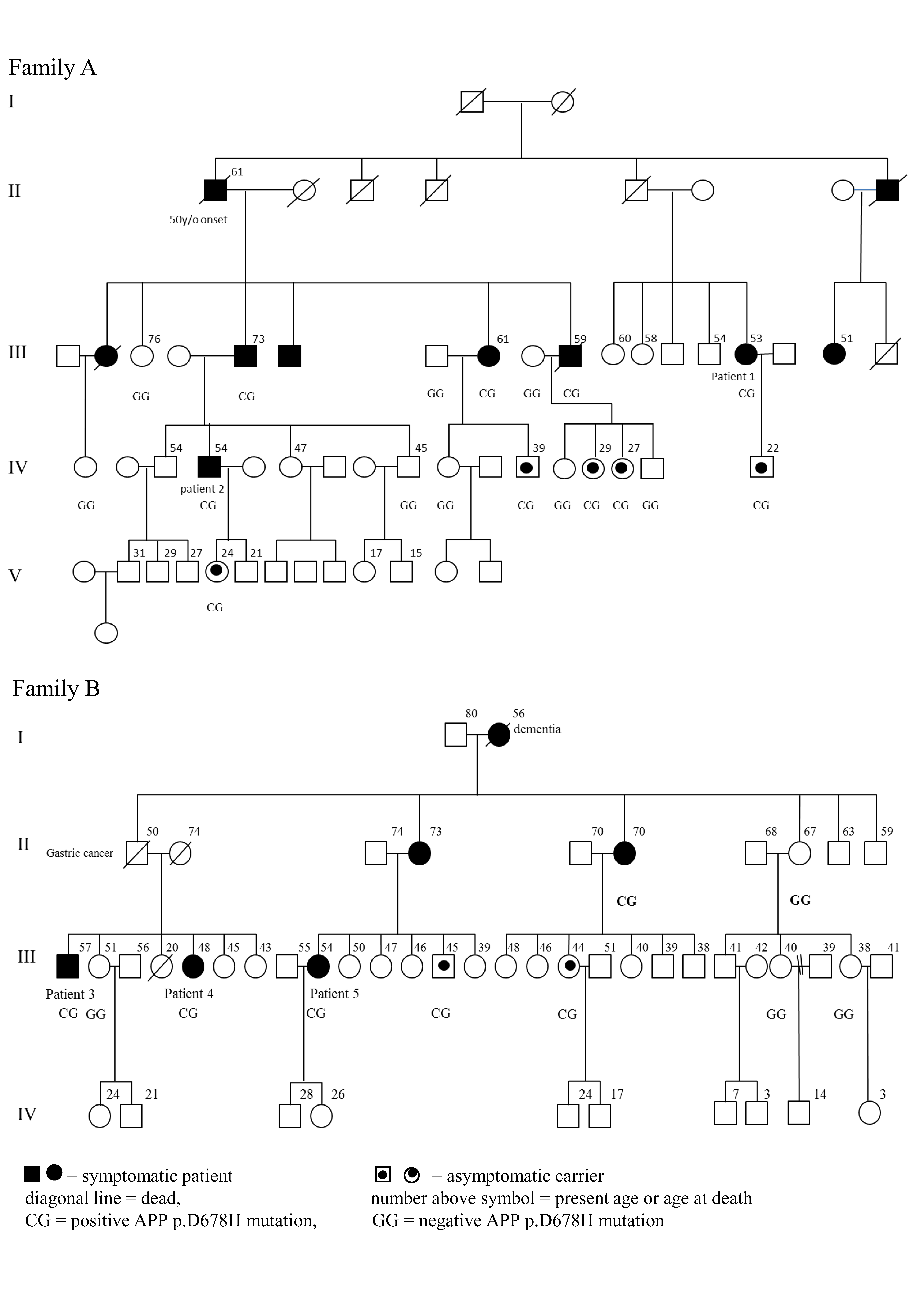

Supplement: Supplementary file 1 [file Image_1.tif]
